# Hydroacoustic sensing of seismic events during the Tajogaite volcanic eruption (La Palma, Spain)

**DOI:** 10.1038/s41598-025-88509-z

**Published:** 2025-02-03

**Authors:** Jesús Alcázar-Treviño, Guillermo Lara, Eduardo D. Suarez, Manuel Bou, Itahiza Domínguez, Susannah Buchan, Francisco Domínguez, Eugenio Fraile-Nuez

**Affiliations:** 1 Centro Oceanográfico de Canarias, IEO-CSIC, Santa Cruz de Tenerife, 38180 Spain; 2 Departamento de Ingeniería Industrial, Universidad de La Laguna, San Cristóbal de La Laguna, 38200 Spain; 3 Centro Oceanográfico de Murcia, IEO-CSIC, Lo Pagan, 30740 Spain; 4 Unidad Mixta IEO-UPV, Gandía, 46730 Spain; 5 Universidad de La Laguna (ULL), San Cristóbal de La Laguna, 38203 Spain; 6 Instituto Geográfico Nacional, Santa Cruz de Tenerife, 38003 Spain; 7 Center for Oceanographic Research COPAS COASTAL, Universidad de Concepción, Concepción, 4030000 Chile; 8 Departamento de Oceanografía, Facultad de Ciencias Naturales y Oceanográficas, Universidad de Concepción, Concepción, 4030000 Chile; 9 Centro de Estudios Avanzados en Zonas Áridas (CEAZA), La Serena, 1700000 Chile; 10 DC Servicios Ambientales, Santa Cruz de Tenerife, 38003 Spain

**Keywords:** Seismology, Marine acoustics, Hydroacoustics, Volcanic eruption, Seismology, Volcanology, Physical oceanography

## Abstract

Volcanic processes generate a variety of seismic events that can be detected by both on-land and underwater sensors. During the 2021 subaerial eruption of the Tajogaite volcano on La Palma Island (Canary Islands, NW Africa), an underwater acoustic sensor was strategically deployed to monitor seismic activity. This study presents marine passive acoustic monitoring data from a moored hydrophone deployed offshore at a depth of 77 m and 7 km from the volcanic vent, both during and after the eruption. We compare hydrophone recordings with island’s seismic network and earthquake database from the Instituto Geográfico Nacional (IGN). By calculating acoustic metrics and analyzing low-frequency bands (< 100 Hz), we identified 712 impulsive acoustic signals consistent with seismic events recorded in the seismic catalogue. These acoustic signals were double-pulsed, low-frequency (≤ 50 Hz with peak frequencies ≤ 15 Hz) and exhibited sound levels that well correlated with earthquake magnitudes. Our findings demonstrate that shallow-water hydro-acoustics can detect and estimate the magnitude of volcano-tectonic earthquakes in the studied scenario. These results encourage for the integration of hydro-acoustic monitoring in conjunction with on-land seismic stations to enhance the overall monitoring of the investigated volcanic area seismic activity.

**Supplementary Information:**

The online version contains supplementary material available at 10.1038/s41598-025-88509-z.

## Introduction

Seismicity associated with subaerial volcanic eruptions is typically monitored using seismic stations placed on land^1,2^, which detect body waves, specifically Primary (P) and Secondary (S) waves^3^. Upon reaching the earth’s crust-ocean interface, a portion of these waves is converted into acoustic waves that travel long distances through the SOFAR (SOund Fixing And Ranging) channel^4,5^. These acoustic waves can eventually reconvert into seismic waves upon re-entering the earth’s crust and are detected as Tertiary or T-waves by on-land seismic stations, following the initial P and S-waves^4,6^.

In oceanic islands, seismic networks are often confined to the landmass, potentially introducing biases in earthquake location analysis due to the limited distribution of sensors of the seismic network. Enhancing seismic network by deploying ocean-bottom seismometers (OBS) can mitigate this issue^7,8^. However, OBS deployment, maintenance, and the data processing and analyses of its three-components are labor-intensive and costly. An alternative is the use of hydrophones, which are less expensive and easier to deploy, maintain and to analyze in the case of single-channel equipment. Hydrophones can record earthquake-related signals both from distant sources via single acoustic T-wave in the SOFAR channel at > 200 m depth (typically between 800 and 1200 m depth)^4,5,9,10^, and from local/regional sources when resting on the seabed, acting as accelerometer to detect P-waves^11,12^.

Passive acoustic monitoring (PAM) is a promising solution for detecting seismicity in remote, hard-to access areas such as oceanic islands. Volcanic archipelagos, like the Canary Islands off NW Africa, frequently experience seismic activity related to magmatic movements and eruptive processes^13,14^. On September 19, 2021, the Tajogaite volcanic eruption started in Cumbre Vieja, La Palma (Canary Islands, Spain), following eight days of precursor activity that included more than 5,000 seismic events^15^. The eruption, which lasted 85 days, produced lava flows that reached the sea^16,17^ while land-based seismic stations were registering earthquakes and tremors associated with the eruption^18^. The lava flows formed two lava deltas in a Special Area of Conservation (SAC) –Franja Marina de Fuencaliente–that is part of the “Natura 2000” network of the European Commission, which stipulates the close monitoring of impacts on the biota and habitats of this area^19^.

As part of the intensive scientific monitoring during the eruption, a hydrophone was deployed to investigate possible changes in the marine soundscape close to the zone of influence of the newly formed Tajogaite volcano. Taking advantage of acoustic data recorded by the monitoring node placed offshore, 1 km from the west coast of La Palma, where the lava deltas were formed, we investigated the geophonic contributions and evolution of the overall marine soundscape within the SAC. Concurrently, land-based seismic stations were recording earthquakes associated with the eruption. Our goal was to coordinate these two independent datasets from different sensors placed underwater and on land to prove that hydrophones close to the seabed record volcano-tectonic earthquake signals like the ones found in land-based seismometers. Moreover, we hypothesized that the sound pressure levels of these water-borne acoustic signals would correlate with the magnitudes of their corresponding seismic signals.

## Results

**Fig. 1:**
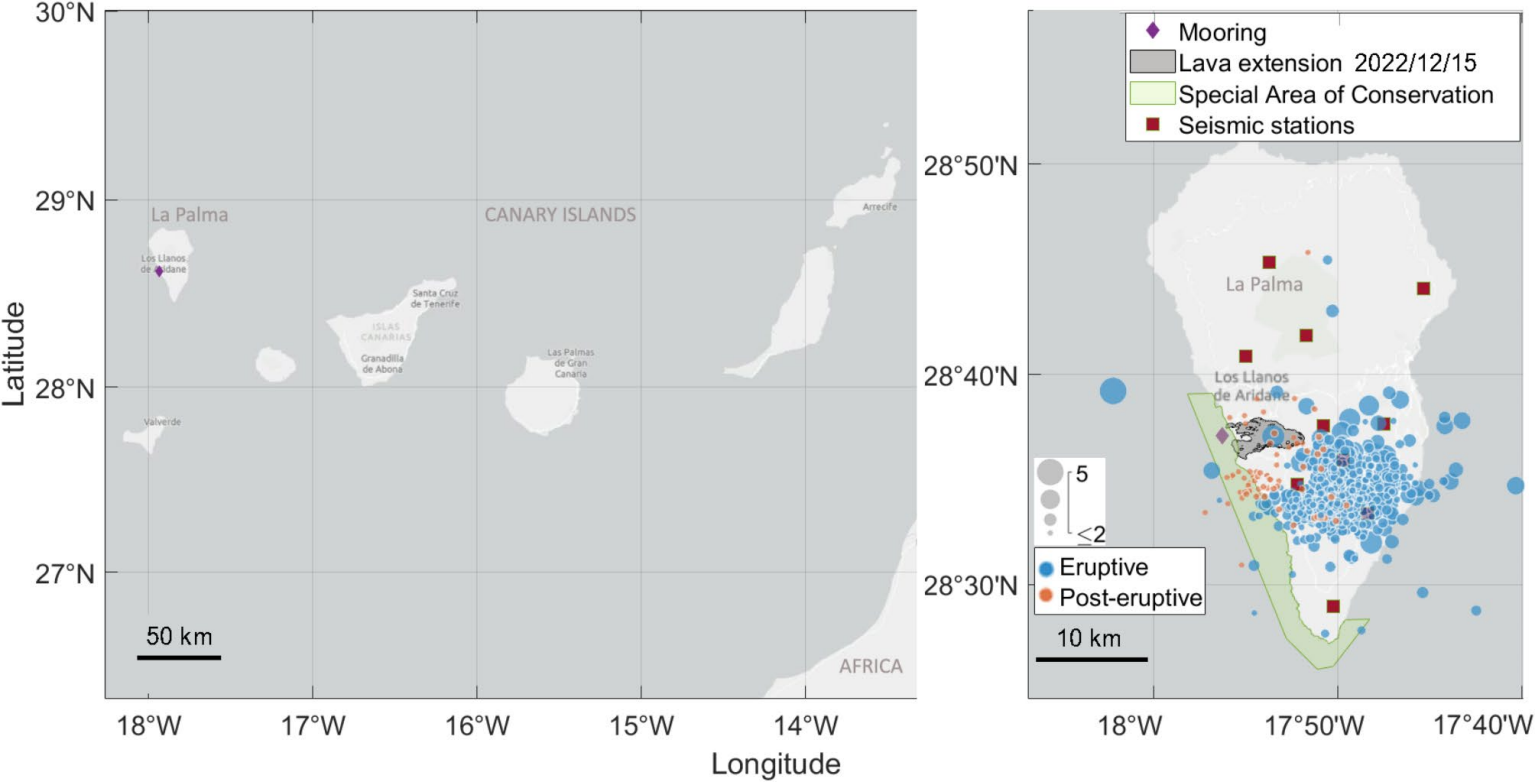
Maps showing the Canary Islands (NW Africa, left panel) and La Palma Island (right panel) key locations and features. The hydrophone mooring location is indicated by a purple diamond. The 10 land-based seismic stations are marked with red squares. The Special Area of Conservation (SAC) Franja Marina de Fuencaliente, is shaded in green. The area covered by lava as of December 15th, based on a post-eruption drone survey, is shown in gray (data from Cabildo Insular de La Palma, https://www.opendatalapalma.es). Earthquakes detected by land-based seismic stations during the hydrophone deployment periods are shown by blue and orange circles. The diameter of each circle corresponds to the earthquake’s magnitude mbLg^20^, with blue circles indicating events during the eruption and orange circles representing post-eruptive detections. The maps were generated using the functions *geoplot* and *geobubble* for MATLAB R2022a (see Methods section).

We are reporting results from two independent datasets that were part of the scientific monitoring of the 2021 eruption at La Palma and a survey one year after the event. These datasets are comprised of the PAM from a hydrophone moored in 77 m water depth close to the newly formed lava deltas (Table 1; Fig. 1) and the data from the land-based seismic monitoring network of the Instituto Geográfico Nacional (IGN)^20^ (see details in Methods section).

**Table 1:** List of hydrophone deployments.

Deployment #	Dates start-end (total days)	End-to-end calibration (dB)	Sampling frequency (kHz)	Depth (m)	Context
1	2021/10/19 2021/10/28 (< 9)	185.8	24	77–78 (2–3 m above the seafloor)	During the eruption
2	2022/12/07 2022/12/10 (< 3)	173.3	288	77–78 (2–3 m above the seafloor)	One year post-eruption, with vessel nearby
3	2022/12/10 2022/12/22 (< 12)	173.3	288	80 (resting on the seafloor)	One year post-eruption, seabed recording

The PAM equipment was installed using a typical mooring line composed by the anchor, acoustic recorder and buoy to reach a vertical geometry and therefore obtain soundscape composition in water column. Soundtrap hydrophone sensitivity was provided by the manufacturer, operating the device at low gain settings (2021) and high gain settings (2022). Special Area of Conservation, La Palma (Longitude: 28.61785 N, Latitude: 17.938143 W, WGS-84).

### Shallow water hydro-acoustic signals

**Fig. 2:**
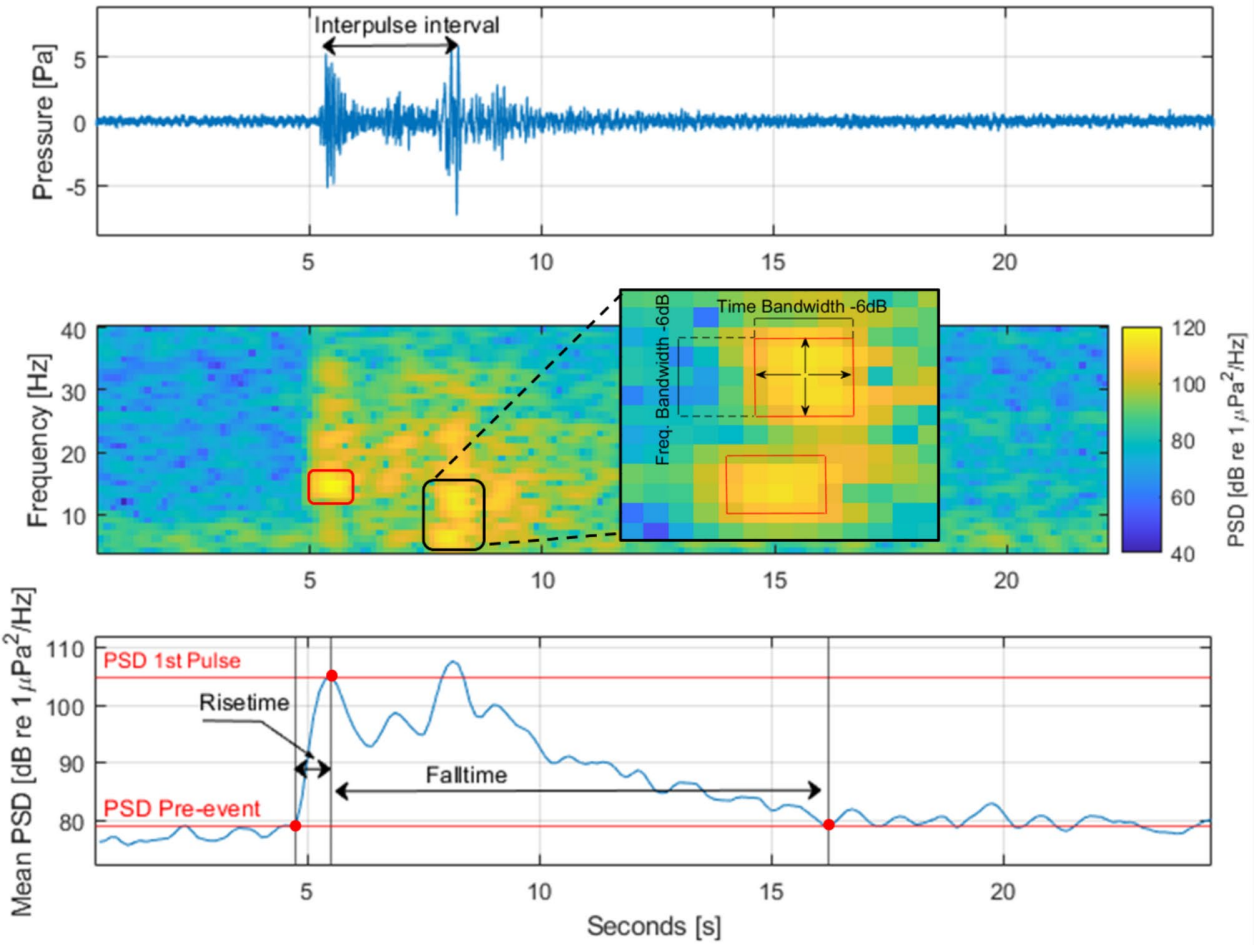
Example of a sound event in the temporal domain (top panel), time-frequency diagram (Power Spectral Density, Nfft = 24,000, overlap of 87.5%, middle panel) and averaging the frequency samples of the PSD in each temporal bin (bottom panel). For this article, Risetimes and Falltimes as shown in the bottom panel have been calculated from the pre-event PSD level and the maximum PSD level of the first pulse of the same event.

A detailed visual examination of low-frequency spectrograms from the PAM data during the first deployment identified a total of 712 impulsive acoustic signals, which were treated as candidate hydroacoustic earthquake detections for characterization. Each signal was characterized by two main pulse (see Fig. 2a). The first pulse was primarily centered at a frequency of 15 Hz, while the second pulse exhibited two frequency components at 6 Hz and 14 Hz (highlighted by red boxes in Fig. 2b). A key defining feature of these sound events was the separation between the two main pulses, known as the inter-pulse interval (Fig. 2a). The parameters of these signals are summarized in Table 2.

**Table 2:** Calculated parameters (main and standard deviations values reported) for the 712 acoustic events classified as hydroacoustic earthquake detections.

Double-pulsed signals	Time bandwidth, − 6 dB (s)		Frequency bandwidth, − 6 dB (Hz)	Peak frequency (Hz)	Inter-pulse interval (s)
**1st pulse**		0.62 ± 0.07	4.22 ± 1.30	15.00 ± 0.79	2.61 ± 0.12
**2nd pulse**	Dataset #1	0.70 ± 0.13	3.01 ± 0.72	6.44 ± 0.64	
	Dataset #2	0.71 ± 0.14	3.92 ± 1.63	13.94 ± 1.82	

Inter-pulse interval is referred to the time period between the two consecutive pulses of each event. We present two sets of measurements for the 2nd pulse according to Fig. 2.b where red squares that show they are better analyzed separately.

Both pulses were typically accompanied by their respective echoes. The maximum amplitude order of the pulses was variable; the first pulse was not always greater than the second one. Additionally, the amplitude of the frequency components in the second pulse varied, with 6 Hz component sometimes being higher than the 14 Hz component.

The duration of the sound (Fig. 2c) was dependent on the intensity of the event. For instance, an event with a 30 dB increase above the preceding PSD level (referred to as PSD Pre Event in Fig. 2c) took longer to return to the baseline (fall time in Fig. 2c) compared to an event with 10 dB increase. This variability in fall time reflects the differing energy levels and recovery times of acoustic events.

**Fig. 3:**
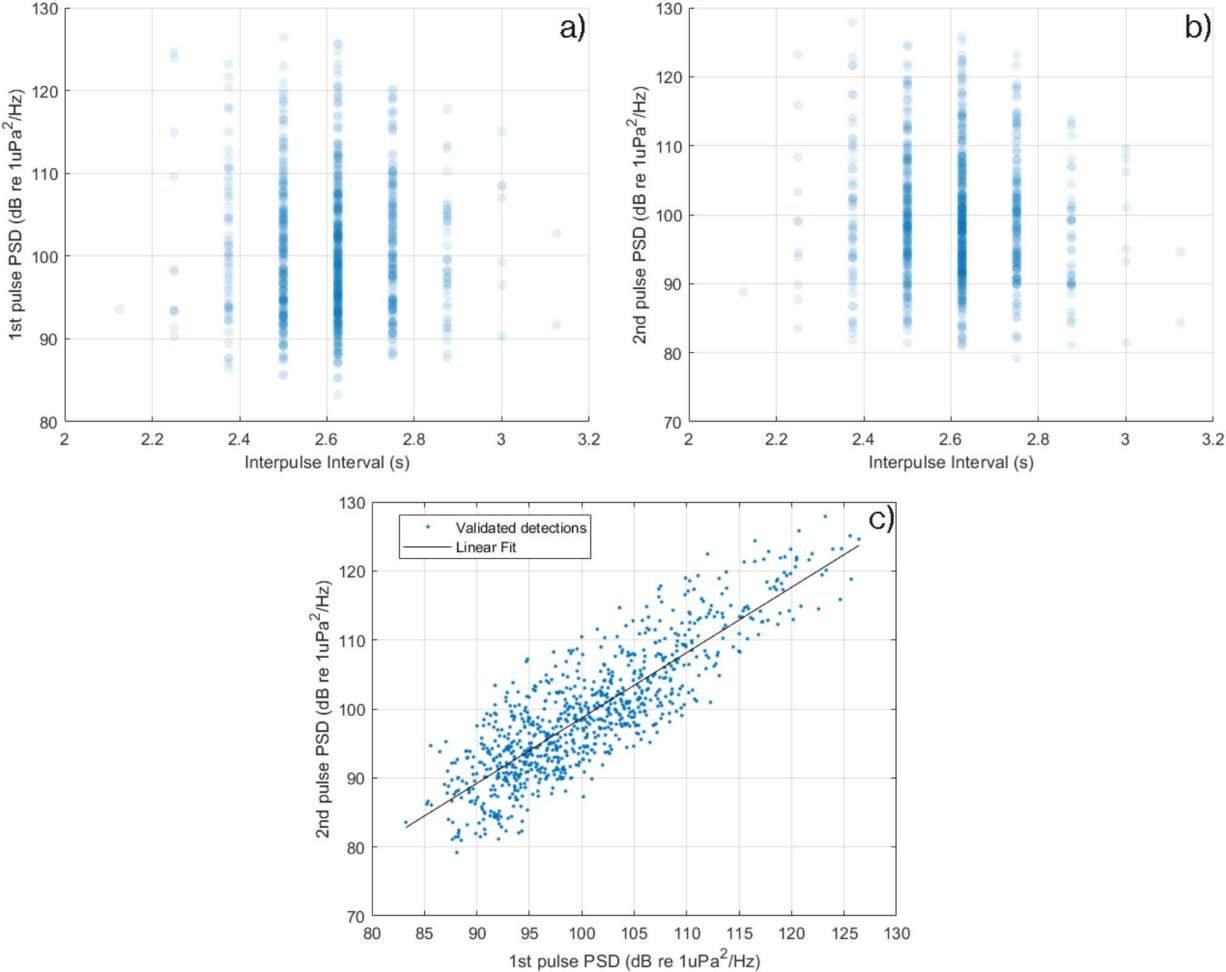
Scatter plot between the PSD’s and the inter-pulse intervals of: (**a**) the first pulses; (**b**) the second ones; each blue point represents a validated signal detection. (**c**) Scatter plot between the PSD’s of the second versus the first pulse of each validated signals detection.

Continuing with the characterization of the double-pulsed signal detections carried out, Fig. 3 shows scatter plots to understand the relationship between the PSD and temporal distance between pulses, specifically, for the first pulse (Fig. 3a), and the second one (Fig. 3b). It is verified how these characteristics are not related, that is, the temporal distance between pulses does not depend on the PSD of any of the two pulses of which the signal is formed.

Furthermore, Fig. 3c represents a scatter plot between the PSD obtained from the first pulse and the second one of each of the validated detections. It can be seen how there is a quasi-linear relationship (y = 0.95·x + 3.99 and R^2^ = 0.89) between both PSD’s, in other words, the energy contained in the dominant frequency in each of the pulses is similar. These results suggest that P-wave (first pulse) tends to have more energy than S-wave (second pulse) in the water-column.

### Seismo-acoustic coupling

**Fig. 4:**
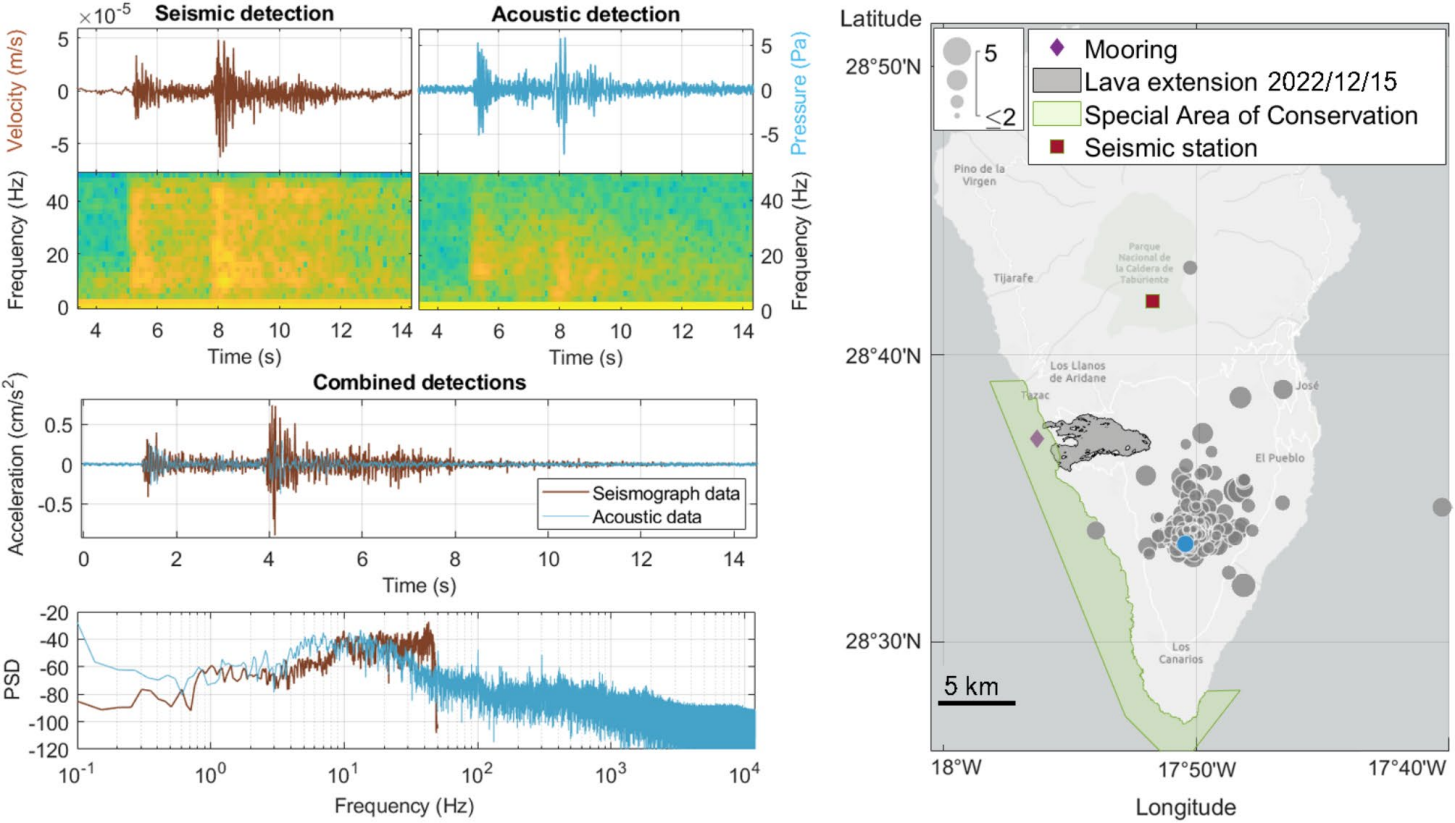
Left multi-panel: comparison of seismic and acoustic data for the same event, aligned on a common temporal axis, showing high temporal and waveform similarities. The vertical scales are velocity (m/s) for the seismic sensors and pressure in Pascals (Pa) for the hydrophone. Both waveform plots are accompanied by a bottom panel showing each PSD signal spectrogram (Nfft = 50 and 12000 for the seismic and acoustic data, respectively, overlap = 80%. Note that the sampling rate for the seismograph was 100 Hz while the hydrophone was set to sample at 24 kHz). The bottom panels show a coordinated waveform for both datasets converted to acceleration (cm/s^2^) and the PSD calculated for the two acceleration signals (as in Iannaccone et al.^11^, see Methods section). Right panel: geographic map showing the epicenters of events in the IGN catalogue that were acoustically detected represented by grey circles except for the example event shown in the left panel, represented in the map as a blue-filled circle. The diameter of each circle corresponds to the earthquake’s magnitude, as indicated in the upper-left legend. The location of the seismic station ‘CMIR’ is also shown on the map. The epicentral distance of the displayed earthquake was at about 12 km to the hydrophone and 16 km to the CMIR seismic station. The map was generated using MATLAB R2022a (see Methods section).

We compared the underwater acoustically detected seismic signals with the seismic catalogue from the land-based seismic data (see Methods), identifying 712 matching signals. A comparative analysis of the data recorded by the land-based seismometers and the moored hydrophone for identical signals revealed high similarities in terms of duration and occurrence of the two main pulses (Fig. 4), confirming the match between both independent datasets.

**Fig. 5:**
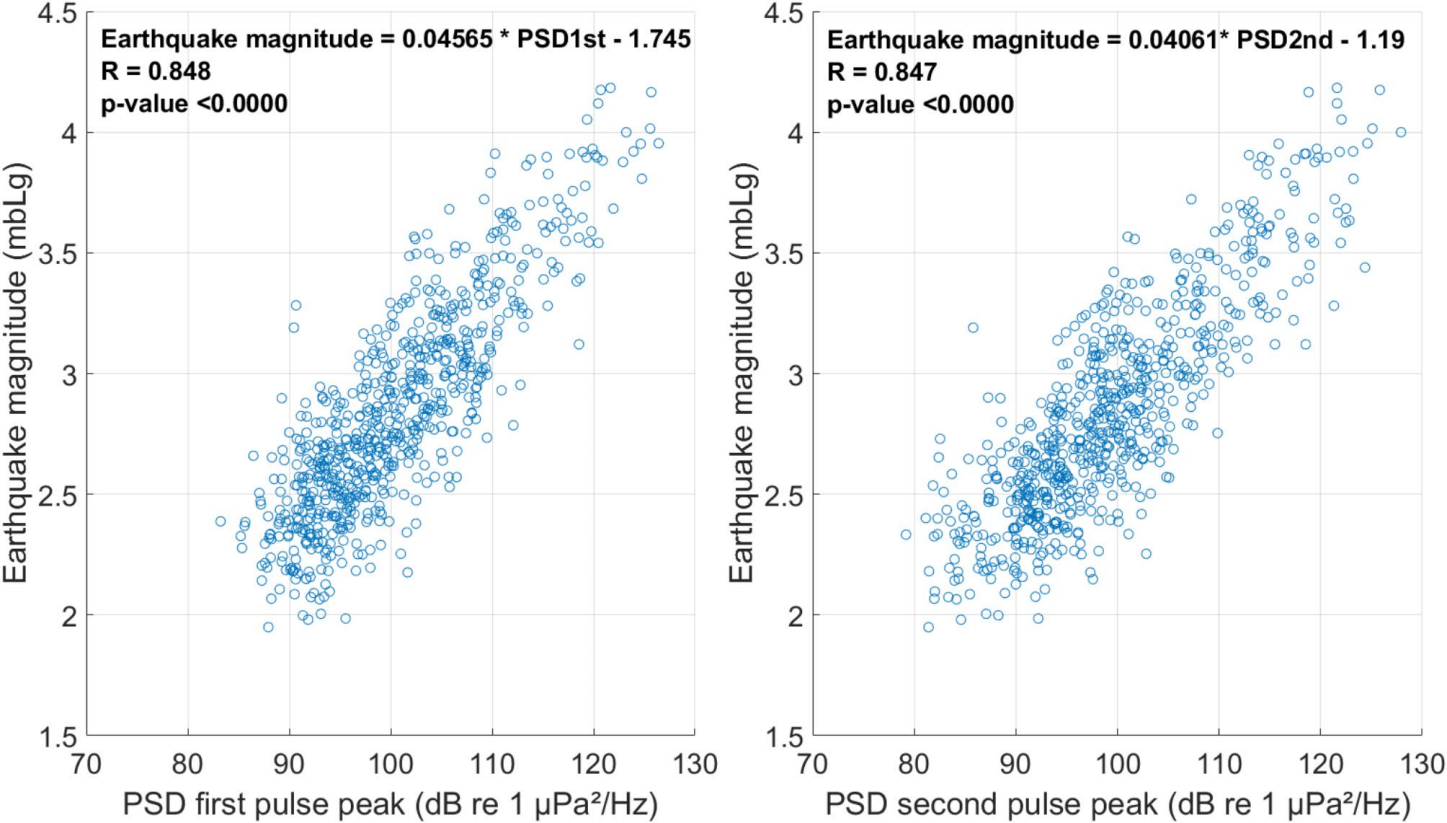
Scatter plots illustrating the hydro-acoustic detections allocated in the seismic catalogue, combining variables from the two independent datasets, i.e.: PSD from passive acoustic monitoring data and earthquake magnitudes from land-based seismic stations. Each plot includes the results from Pearson Correlation tests, displaying the correlation equation, the correlation coefficient (R), and the p-value.

The coupling between seismic waves traveling through a solid medium and acoustic waves in the water column means a potential relationship between variables measured from both systems. In order to explore this possibility, we plotted the power spectral density (PSD dB re 1 µPa^2^/Hz) of the two main peaks of the acoustic signals against the earthquake magnitude (mbLg, see Methods section) calculated from the land-based seismic station data. This analysis revealed a good agreement (zero p-value and *R* = 0.84, see Fig. 5), further validating the assignation of acoustically detected signals to events in the seismic catalogue.

### Overall marine soundscape

**Fig. 6:**
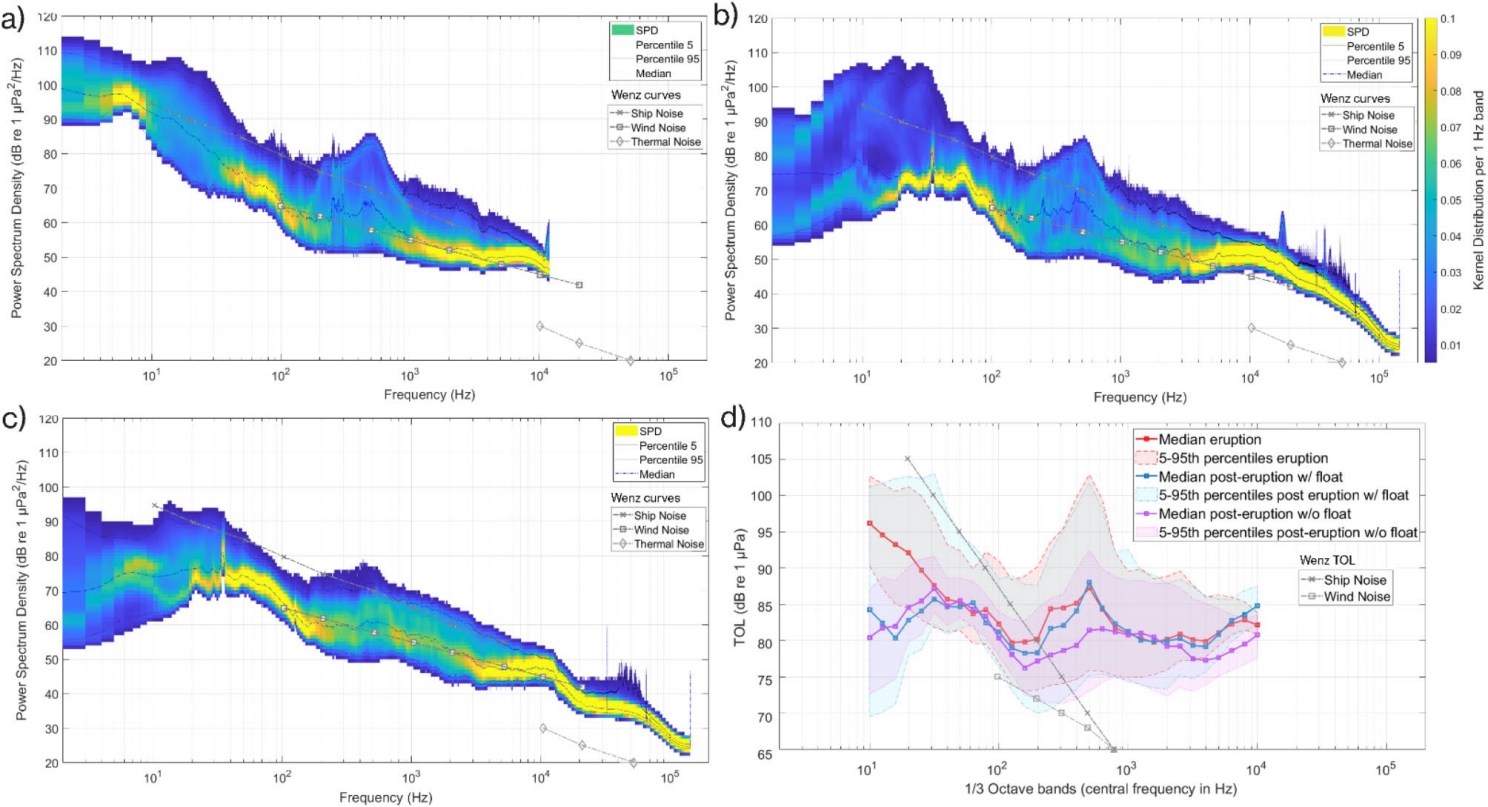
PSD plots colored by their Kernel spectral probability distribution (SPD, color bar) for the (**a**) first deployment, during the volcano eruption; (**b**) second deployment, after the eruption with the hydrophone moored in the water column and the vessel working nearby; (**c**) third deployment, after the eruption with the hydrophone on the seabed; (**d**) TOLs (1/3 Octave band sound pressure levels) showing median values (solid lines) and 5-95th percentiles (colored shade) for the first (red), second (blue) and third (purple) deployments. Wenz curves were added as gray lines in both PSD and TOL plots (see Methods section). Notice that the Thermal Noise curve is not shown in the TOL plot since the values were below our TOL results.

We represented the overall soundscape during the three hydrophone deployments by calculating median values of PSD for each 60 s time-interval of recordings (see Methods). Kernel probability distribution values per 1 Hz were used to color-code the data. This visualization (Fig. 6) provided an at a glance overview of soundscape, revealing an apparent bimodal distribution in PSD values for the lower frequencies (< 100 Hz) and some medium frequencies (around 500 Hz) across all the three deployments. Biophonies such as evening fish choruses are compatible with higher sound levels at 500 Hz^21–23^.

Focusing on the low frequencies (< 100 Hz), where acoustic signals of volcanic-seismic events could be detected, we observed that the first deployment had clearly higher PSD values (Fig. 6a). One-third octave received sound levels (Third Octave Level, TOL dB re 1 µPa) were calculated for all deployments and plotted together (see Methods). The median TOLs for low frequencies were noticeably higher during the first deployment (Fig. 6b), which coincided with the Tajogaite volcanic eruption, compared to the post-eruption measurements (second and third deployments, Fig. 6b and Fig. 6c, respectively). During these post-eruption periods, the hydrophone was either deployed in the water column (second deployment) or resting on the seabed (third deployment). The median values for the second and third deployments were very similar, as were their lower percentile values, despite the different recording set up (Table 1).

The first and second deployments had similar TOL values in their upper percentile (95th percentile), particularly when a vessel was operating nearby during the second deployment. In contrast, the upper percentile values were lower for the third deployment, when no vessel was present.

## Discussion

During the subaerial eruptive process that resulted in the formation of the Tajogaite volcano in 2021, nine days of continuous PAM data captured 712 low-frequency impulsive acoustic signals. The signals exhibited a characteristic pattern of two consecutive pulses at ≤ 15 Hz (Fig. 2; Table 2). Regarding the spectral characteristics, these signals can be classified as high-frequency volcano-tectonic earthquakes (HF, VT or A-type), with dominant frequencies in the 5–15 Hz range^3^, in alignment with the classification from seismic data recorded by land-based stations network.

The double-pulsed nature of the acoustic signals and their stable inter-pulse interval of 2.6 s closely matched the metrics extracted from seismic recordings taken during the same period from land-based stations^18^. This congruence between datasets highlights the potential for using hydroacoustic data to complement traditional seismic monitoring. In this study, these two independent datasets showed a strong positive correlation, with 712 acoustic events detected by the hydrophone coinciding with the automatic seismic catalogue obtained from the IGN network. These coinciding detections allowed us to compare waveforms from the two independent datasets which showed a greater similarity than expected (Fig. 4). Hydrophones deployed in the water column have been previously reported to capture T-wave from distant earthquakes traveling through the SOFAR channel, typically showing a single signal in the waveform^4,5,9,10^. When deployed on the seabed, hydrophones can act as accelerometers, recording the same earthquake signals as a collocated seismic station^11,12^. However, our study presents a novel scenario where a hydrophone deployed near the seafloor in shallow water (< 100 m), recorded signals highly similar to those concurrently registered by a land-based seismic station located at about 12 km away (Fig. 4), capturing signals resembling both P and S-waves. This finding suggests a more efficient coupling between the seismic and hydroacoustic systems of the lithosphere-ocean interface than previously theorized near the seabed^10,24,25^. Our results suggest that a hydrophone floating close enough to the seabed (2–3 m) is able to register both P and S-waves and therefore the hydrophone would also function as an accelerometer for the low frequency bands in this arrangement. However, the acoustic recording lacks the earthquake high frequency components found in the seismic record (Fig. 4) as it was previously reported for records at similar depths^11^. Moreover, since the first pulse in the acoustic signals is normally louder than the second one (Fig. 3c), the acoustic data recorded some 2–3 m above the seabed is probably showing how rapidly the S-wave is decaying in the water-column. This is corroborated by comparing acceleration waveforms for the same earthquake obtained from the seismic record and the hydrophone data (Fig. 4). Here, the P-wave or first pulse is almost identical in magnitude between sensors, but the S-wave is clearly smaller in the acoustic recordings, probably because of its rapid loss of energy in the layer of water closest to the seabed.

The coupling between the solid-traveling seismic waves and the water-borne ones is further supported by the positive correlation between hydroacoustic and seismic data reported for this study (Fig. 5). This correlation allowed us to estimate the approximate magnitude of these volcano-tectonic earthquakes by calculating the sound pressure level as PSD of one of the two signal peaks. Previous studies have done this for T-waves travelling in the SOFAR channel at depth greater than 200 m^9^ and for hydrophones deployed on the seabed acting as accelerometers^12^. However, to our knowledge, this is the first time this correlation has been demonstrated for a hydrophone deployed close to the seabed in shallow waters during an ongoing eruptive process.

This innovative finding has the potential to enhance seismic monitoring before, during and after an eruption. Deploying an array of hydrophones in the shallow coastal areas could complement existing networks of land-based seismic stations. Furthermore, the predictions from this positive correlation could be the basis of a proxy for the acoustic contribution of numerous seismic events to the soundscape of surrounding shallow waters.

Our results show a clear difference in the marine soundscape near the new lava deltas between the volcanic eruption (first deployment) and one year later (second and third deployments). The low frequencies (< 100 Hz) exhibited more divergent PSD and TOLs between recordings taken during and after the eruption (Fig. 6). Although biophonies, such as baleen whale vocalizations and some fish sounds can occupy these low frequencies and sometimes explain seasonal variation at low frequency band sound levels^26,27^, we found no evidence of a continuous biological sounds emissions that could explain such a drastic contribution to the TOL metrics of the soundscape.

Human marine activities, such as vessel traffic, also produce sounds in the low frequency bands^28,29^ and contribute to the soundscape more continuously compared with more seasonal or occasional biological sounds^26^. Apart from vessel activity in the area and occasional biological sounds, part of these higher TOLs at low frequencies could be related to the seismo-volcanic activity (e.g. seismic tremor or volcano-tectonic earthquakes) taking place during the eruption. In this regard, the low frequency impulsive signals we described were only present in the recordings during the Tajogaite volcanic eruption (1st deployment, Figs. 2 and 5). Although the land-based seismic stations detected seismicity one year after the eruption, i.e.: concurrently to the 2nd and 3rd hydrophone deployments, we found no signal nor any apparent contribution of these earthquakes to the low frequency bands of the soundscape (Fig. 6). During these 2nd and 3rd deployments, all the earthquakes recorded in the land-based seismometer were of magnitude ≤ 1.9 (see Supplementary Material). The hydrophone in the configuration and scenario we presented could detect hydroacoustic-seismic signals produced by earthquakes with magnitudes > 1.94 (Fig. 5) which seems to be the trigger for the acoustic detection in our analyses. This is because the seismicity after the event was undetected by our acoustic sampling and only recorded by land-based stations, with a mean and maximum magnitudes of 1.28 ± 0.32 and 1.90, respectively. These results should be taken into account for future analyses in this area and hydrophone configuration. They also support findings by previous authors that hydroacoustic monitoring can detect relatively low magnitude events, e.g. Helffrich^10^ who found that events as low as 2.0 magnitude can be hydro-acoustically detected and located.

We propose expanding hydrophone deployments in shallow waters around volcanic islands to increase monitoring and research efforts regarding the seismicity associated with magmatic movements and potential volcanic processes. This would add a valuable layer of information to land-based seismic stations, serving both as an early response mechanism and aiding post-eruption seismic analyses. To ensure coordinated sampling with the seismic network, these hydrophone deployments should include GPS connectivity and precise time-accuracy, transmitting low-frequency data to a land-based receiver for real-time calculations combined with the seismic on-land network. Moreover, coordination with biodiversity monitoring efforts should be made, since earthquakes have been reported to alter cetacean behaviour^30,31^ and the Canary Islands hold the highest cetacean diversity within the European Union^32^.

## Conclusions

This study demonstrates for the first time that a hydrophone deployed in the water column near the seabed at shallow depths (< 100 m) can effectively record seismic events, showing comparable signals as the ones detected with data from a land-based seismic station located within 12 km distance. The reported signals, characterized by double pulses, were derived from volcano-tectonic earthquakes. There was a good correlation between the sound pressure level of each acoustic pulse and the earthquake magnitudes calculated from land-based seismic data.

Our detailed characterization of these signals provides the basis for future PAM efforts under similar configurations and contexts, enabling the development of detection algorithms for acoustics signals related to eruptive processes. Moreover, these signals seem to contribute significantly to the low-frequency bands of the shallow water soundscape near the newly formed subaerial volcano Tajogaite, potentially affecting marine biodiversity in the area sensitive to these frequencies, such as baleen whales that communicate using low-frequency sounds. Deploying an array of hydrophones in shallow waters, surrounding volcanic islands like La Palma, could greatly enhance the monitoring and research of seismic activity. This approach would provide critical data to inform decision-makers, facilitating organized early responses to potential eruptions and posterior analyses of the magnitude of these events. Such an integrated monitoring system could improve the overall understanding and management of volcanic and seismic hazards in oceanic island and coastal environments.

## Methods

### Monitored area and experimental setup

Passive acoustic monitoring was carried out with a 300 STD SoundTrap digital hydrophone, with internal 16-bit SAR analog to digital converter, from OceanInstruments (http://www.oceaninstruments.co.nz/) during three distinct monitoring periods (Table 1). During the first deployment, the hydrophone was configured with a sampling rate of 24 kHz recording continuously. For the subsequent two deployments, the hydrophone was configured with a sampling frequency of 288 kHz. In all three deployments, the hydrophone was moored at the same location, at approximately 77 m depth in a total water column of 80 m, near the coast of La Palma, 2.38 km southeast of Tazacorte harbor and 7.06 km from the newly formed Tajogaite volcano (Fig. 1).

The first deployment coincided with the active phase of the Tajogaite volcanic eruption. The second and third deployments were conducted one year after the eruption at the same location of the first deployment. During the second deployment, an oceanographic vessel was operating nearby. However, the mooring lost its float, causing the hydrophone to lie directly on the seabed for 11 days without the presence of the vessel. This situation was analyzed as a separate, third deployment due to the change of recording setup and context. Across all three deployments, a total of about 24 days of acoustic recordings were analyzed.

Data from the permanent land-based seismic monitoring network of the Instituto Geográfico Nacional (IGN), comprising 10 broadband seismometers on La Palma Island^20^ (Fig. 1), were used for comparative analysis. The CMIR seismographic station shown in Fig. 1 has three components with a sampling rate of 100 Hz^20^. Earthquakes detections from this seismic network were incorporated into the earthquake catalogue and used for the comparative analyses, with magnitude values reported in mbLg^20^.

### PAM data processing

Acoustic recordings were analyzed using the Matlab R2022a (The MathWorks Inc.) framework. For each analysis, acoustic data was calibrated using the SoundTrap end-to-end calibration information provided by the manufacturer (see http://www.oceaninstruments.co.nz).

Since most underwater sounds produced by geological events occur at low frequencies of < 100 Hz^33–36^, we used custom scripts for Matlab to visually examine the recordings in search of low-frequency impulsive sounds described for earthquake detections^4,6^. Once we found and annotated candidate sounds for seismic-hydroacoustic earthquake detections, i.e. recurrent double-pulsed impulsive sounds at < 100 Hz, we characterized the detected signals by studying their pulse duration, inter-pulse interval, peak frequency and temporal occurrence. The signal searching and selection process was made using the internal clock of the digital hydrophone. Then, a visual comparison of the portion of candidate signals for the event comparisons between acoustic and seismograph instruments was done without the use of an absolute time-stamp reference (like GPS), actually not available in our experimental setup. However, we strongly recommend using GPS or equivalent stable clock sources for this kind of research in the future.

In order to show the complete registered soundscape per deployment and the potential contribution of seismic signals to the shallow water soundscape, we calculated PSD values for 60 s windows with a frequency resolution of 1 Hz and 50% overlap. We then calculated Kernel distribution^37^ of PSD values for each 1 Hz band and represented it along the median and 5-95th PSD percentiles. To compare specific soundscape metrics between the three deployments, we calculated one-third octave received sound levels (Third Octave Level, TOL dB re 1 µPa) from 10 Hz to 10 kHz bands of the three deployments for every minute of acoustic data and each one-third octave band. Percentiles 5, 50 and 95 were calculated for each of the three deployments and plotted together for comparison. For both PSD and TOL plots, approximations of the Wenz curves were added to compare our results with estimates of the typical ship, wind and thermal noise present in the Ocean^38^.

### Land-based seismic data

The earthquakes registered in the IGN seismic network during La Palma 2021 volcanic eruption are publicly available from the IGN bulletin^20^. However, these earthquakes were obtained and revised by analyst during the daily monitoring. Due to the increased seismic activity during the volcanic eruption, only well-recorded earthquakes were included in the seismic catalog, and many low magnitude earthquakes were discarded. To address this, we analyzed the seismic activity in October 2021 to obtain a refined dataset by template-matching using the EQcorrscan module^39^ and the IGN bulletin earthquakes as templates. The new seismic catalog contained 4832 earthquakes that we used to compare with our acoustic records.

### Analyses of two independent datasets

To compare both sets of independent data, i.e. seismic and hydroacoustic earthquake detections, we had to assign the acoustic detections made from the hydrophone recordings to events in the IGN automated earthquake catalogue. Since acoustic recordings had a time lag with respect to seismic data due to the deployment configuration, we searched for signal coincidences in the earthquake catalogue within 40 s of the estimated lag time. We then analyzed only accurate detections where there was a single seismic event matching the acoustic signal, discarding both unmatched and double-matched signals. In order to properly compare these two independent datasets, we converted both into acceleration measurements for the earthquake signals. Velocity measurements from the seismograph (cm/s) were converted into acceleration units (cm/s^2^) using a 0.01 s time interval. For hydrophone data we followed a previous study performed at similar depths^11^ and used deployment depth and water density calculated from CTD measurements from a previous study during the eruption^40^ to convert pressure (Pa) into acceleration (cm/s^2^). After the time-correction and conversion into acceleration units (cm/s^2^), concurrent acoustic and seismic data were plotted to characterize the earthquake signals in both domains. Once we had allocated the acoustic earthquake detections in the seismic catalogue, we explored the possibility of predicting earthquake magnitude in the studied scenario via its potential correlation with PSD of the peak of each pulse of the acoustic signal using Pearson’s Correlation test.

## Electronic supplementary material

Below is the link to the electronic supplementary material.

Supplementary Material 1

## Data Availability

The seismic records are available through the public Spanish Digital Seismic Network: doi: https://doi.org/10.7914/SN/ES. Due to their size, acoustic recordings can be made available upon request. The data of hydroacoustic events allocated in the seismic catalogue can be accessed in the Supplementary Material.
